# Deep plasma and tissue proteome profiling of knockout mice reveals pathways associated with *Svep1* deficiency

**DOI:** 10.1016/j.jmccpl.2025.100283

**Published:** 2025-01-10

**Authors:** Colleen B. Maxwell, Nikita Bhakta, Matthew J. Denniff, Jatinderpal K. Sandhu, Thorsten Kessler, Leong L. Ng, Donald J.L. Jones, Tom R. Webb, Gavin E. Morris

**Affiliations:** a Department of Cardiovascular Sciences and NIHR Leicester Cardiovascular Biomedical Research Unit, Glenfield Hospital, Leicester LE3 9QP, UK; b Leicester van Geest multiOMICS Facility, Hodgkin Building, University of Leicester, Leicester LE1 9HN, UK; c Department of Cardiology, German Heart Centre Munich, Technical University of Munich, 80636 Munich, Germany; d Leicester Cancer Research Centre, RKCSB, University of Leicester, Leicester LE2 7LX, UK

**Keywords:** SVEP1, Proteomics, Liquid chromatography-mass spectrometry, Knockout mouse, Deep phenotyping, Cardiovascular disease, Extracellular matrix

## Abstract

Despite strong causal associations with cardiovascular and metabolic disorders including coronary artery disease, hypertension, and type 2 diabetes, as well as a range of other diseases, the exact function of the protein SVEP1 remains largely unknown. Animal models have been employed to investigate how SVEP1 contributes to disease, with a focus on murine models exploring its role in development, cardiometabolic disease and platelet biology. In this study, we aimed to comprehensively phenotype the proteome of *Svep1*^+/−^ mice compared to wild-type (WT) littermates using liquid chromatography-tandem mass spectrometry (LC-MS/MS) bottom-up proteomics in plasma, heart, aorta, lung, and kidney to identify dysregulated pathways and biological functions associated with *Svep1* deficiency. Our findings reveal that *Svep1* deficiency leads to significant proteomic alterations across the mouse, with the highest number of dysregulated proteins observed in plasma and kidney. Key dysregulated proteins in plasma include upregulation of ADGRV1, CDH1, and MYH6, and downregulation of MTIF2 and AKAP13 which, alongside other proteins dysregulated across tissues, indicate disruption in cell adhesion, extracellular matrix organisation, platelet degranulation, and Rho GTPase pathways. Novel findings include significant enrichment of complement cascades in plasma, suggesting dysregulation of innate immune responses and hemostasis due to *Svep1* deficiency. Pathways related to chylomicron assembly and lipid metabolism were also enriched. Additionally, we developed a high-throughput quantitative targeted LC-MS/MS assay to measure endogenous levels of murine SVEP1. SVEP1 was detectable in lung homogenate and showed a significant reduction in SVEP1 levels in *Svep1*^+/−^*vs.* WT, but was not identified in plasma, heart, aorta, or kidney, likely due to expression levels below the assay's detection limit. Overall, this deep phenotyping study provides insight into the systemic impact of *Svep1* deficiency.

## Graphical abstract

**Figure:**
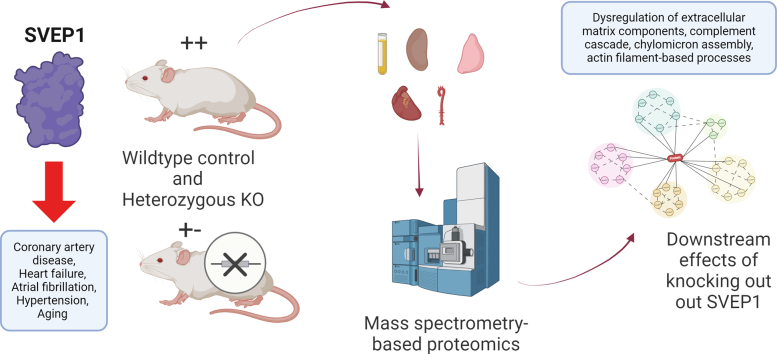

## 1 Introduction

Sushi, von Willebrand factor type 1, EGF and pentraxin domain containing 1 (SVEP1) is a large secreted extracellular matrix glycoprotein that has been identified as associating with several diseases at both the genetic and protein level. First discovered by Shur et al. in 2006, SVEP1 has an unusual composition of functional domains: sushi (complement control protein CCP), von Willebrand factor type A, epidermal growth factor-like (EGF), and pentraxin [1]. Two highly conserved missense polymorphisms in SVEP1 exhibit significant genetic associations: rs111245230 (p.D2702G) associates with coronary artery disease (CAD) [2], BP [2], and elevated SVEP1 plasma levels [[3], [4], [5]]. A second coding variant, rs61751937 (p.R229G) is associated with increased platelet reactivity [6], reduced platelet count (PC) and increased mean platelet volume (MPV) [6], open-angle glaucoma [7], and exerts the strongest effect on SVEP1 plasma levels [[3], [4], [5]], Elevated levels of plasma SVEP1 have been found to associate with several chronic age-related diseases, including aging [8,9], glaucoma [7], pulmonary hypertension [10], incident CAD [5,11] heart failure [11], atrial fibrillation [12], and dementia [13]. Mendelian randomization (MR) analyses indicate causal associations between higher plasma SVEP1 and type 2 diabetes (T2D) [4,5], platelet traits [14], CAD [4], hypertension [4] and dementia [13].

SVEP1 is widely expressed in various tissues including the vasculature [4,[15], [16], [17]], adipose [15], bone marrow [18,19], lung [20], and placenta [21]. SVEP1 has been shown to bind to integrin α9β1 [20], platelet and endothelial aggregation receptor-1 (PEAR1) [14], and tyrosine-protein kinase receptor-1 (TIE1) [22]. Interactions with other receptors, including integrin α4β1 [23,24], angiopoietin 1 (ANG1) and ANG2 [16] and the Notch family [4] have been identified. Given its size and varied domain structure composition, it is likely that SVEP1 also interacts with additional unidentified binding partners, providing the potential for diverse downstream pathway activation. The wide expression profile and localization of SVEP1 across several tissues and in plasma, and the numerous genetic and aptamer-based proteomic studies provide strong evidence of the involvement of SVEP1 in disease, in particular cardiovascular and metabolic disorders, however the mechanisms underlying these associations remain poorly understood.

Animal models have been employed to investigate how SVEP1 contributes to disease, with a focus on murine and zebrafish models exploring its role in development, cardiometabolic disease and platelet biology, reviewed elsewhere [25]. Perinatal mortality is observed in *Svep1* null mice, with embryos lacking valve development, lymphatic remodeling, and exhibiting oedema [16,17]. Heterozygous Svep1 mice display reduced SVEP1 gene expression in several organs, including aorta and lung [15]. *Svep1* deficiency alters lymphatic development in mice [16,17,26], whilst in zebrafish, *Svep1* deficiency causes blood vessel anastomosis [27] and modulates epidermal differentiation [28]. Conditional *Svep1* knockout models have been used in platelet studies, revealing alterations in platelet activity in response to ADP [14], and increased white and red cell blood counts [14]. Mouse models have been instrumental in studying cardiometabolic diseases including CAD and hypertension, with *Svep1* deficient mouse models yielding varying outcomes. Jung et al. found that atheroprone *Svep1*^+/−^*Apoe*^−/−^ mice fed a high-fat diet had a 2-fold increase in SVEP1 expression relative to control mice [4]. Conversely, Winkler et al. found an increase in plaque size associated with *Svep1* deficiency [15].

Given the inconsistencies in atherosclerotic studies, and the wide range of diseases that can be modelled using *Svep1* deficient models, the global characterization of the *Svep1*^*+/−*^ knockout mouse model would provide a beneficial resource. Advances in high-throughput analyses of the proteome offer promise in unravelling the complexity of downstream protein networks influenced by *Svep1* expression. We sought to infer pathways and biological changes associated with *Svep1* deficiency by measuring downstream impacts to the phenotype of *Svep1*^*+/−*^ mice with liquid chromatography-tandem mass spectrometry (LC-MS/MS) bottom-up proteomics. Shotgun proteomics offers a broad proteotyping approach to investigate the function of genes beyond terminal test phenotyping alone; it is protein expression that principally determines the function of cells [29]. Herein we employ deep proteome profiling with high pH reversed-phase fractionation of the plasma of *Svep1*^*+/−*^ mice and wild type (WT) littermates to measure differential expression of proteins and deduce which pathways are impacted with gene ontology (GO) pathway enrichment analysis. Subsequently we sought to further explore and validate these altered systemic plasma protein profiles with a multi-organ tissue study comparing the heart, aorta, lung and kidney proteome of *Svep1*^*+/−*^ mice to WT counterparts, providing a comprehensive overview of the protein-level phenotype of *Svep1*^*+/−*^ mice.

An additional consideration in the study of the contribution of SVEP1 to disease is the measurement of the SVEP1 protein. Previous work measuring SVEP1 in human plasma has relied on data collected using the SomaScan aptamer platform in large-scale studies such as the INTERVAL study [3], or the use of antibodies in many smaller studies. However, SomaScan is designed to measure large multiplexed protein panels and is not a practical or accessible option for many smaller functional studies, and some authors have found SVEP1 antibodies to be unreliable [30]. Therefore, reliable measurement of the protein in biological systems can be an onerous task. In many studies, indirect measurements of expression through RNA are used [4,15,28], or other elegant solutions have been devised such as use of the recombinant protein as a bait protein for proximity-based experiments [30]. LC-MS/MS has the potential to enable an alternative approach; in addition to allowing system-wide profiling of the proteome, it has the potential to be utilized for measurement of SVEP1 in biological systems. Murine SVEP1 has previously been measured in a shotgun mass spectrometry-based draft of the mouse proteome by Giansanti et al. [31], however due to the stochastic nature of untargeted LC-MS/MS data acquisition, differences in experimental conditions, and varying instrument sensitivity, the detection of SVEP1 in all shotgun MS experiments is not guaranteed. Thus, we aimed to overcome the difficulties associated with directly measuring SVEP1 in biological systems by developing a high throughput, quantitative targeted multiple reaction monitoring (MRM) LC-MS/MS assay to measure endogenous levels of murine SVEP1.

## 2 Methods

### 2.1 Mouse tissue and plasma collection

**Fig. 1:**
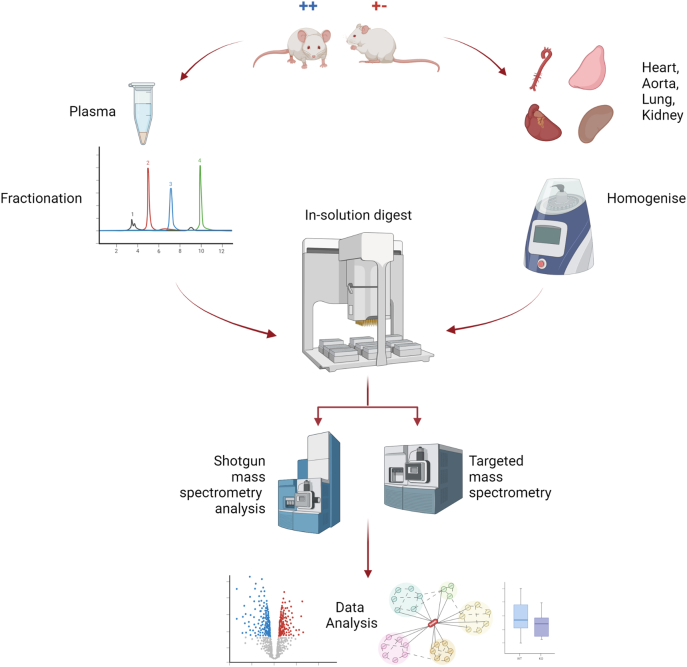
Flow diagram showing the workflow followed in this work. Plasma, heart, aorta, lung and kidney was harvested from *Svep1*^+/−^ and WT littermates. Samples were prepared using automated bottom-up proteomics protocols with the Bravo AssayMAP, with plasma subjected to fractionation to provide deeper proteome coverage. Samples were analysed by shotgun mass spectrometry to provide phenotyping of the downstream pathways affected by *Svep1* deficiency, and by targeted mass spectrometry assay for unique SVEP1 peptides.

All animal care and experimentation were approved by the local animal ethics committee, and performed according to ARRIVE (Animal Research: Reporting of *In Vivo* Experiments) guidelines [32], under the United Kingdom Home Office Project License (P4E9A1CCA). All mice were housed in a specific pathogen-free facility in an individually ventilated caging system, with mice group housed wherever possible, and their health status was checked routinely. This study was performed as an exploratory study using surplus animals from breeding for other SVEP1 studies. No mice exhibited any adverse effects. C57BL/6 J mice were purchased originally from Charles River, then bred in the Preclinical Research Facility in the University of Leicester. Genetically altered animals, B6N(Cg)-Svep1tm1b(EUCOMM)Hmgu/J (*Svep1*^*+/−*^) were purchased from the Jackson Laboratory (Bar Harbor, ME, USA). In accordance with Schedule 1 of the Animals (Scientific Procedures) Act 1986 (U.K.), mice aged between 8 and 25 weeks of both genders were anaesthetized with 4 % isoflurane in medical O_2_. Blood was extracted through cardiac puncture into 3.2 % citrate buffer. Blood was centrifuged at 3000*g* for 10 min, and plasma was extracted and then centrifuged at 3000*g* for a further 10 min, with plasma then stored at −80 °C until use. Tissues were extracted from the mice and stored at −80 °C until homogenization. Fig. 1 shows a workflow diagram for the sample preparation and analysis performed in this work.

### 2.2 Tissue homogenization

This work was performed as an exploratory study using surplus animals from breeding for other SVEP1 studies, thus sample sizes were based on animal availability. Tissues were selected based on both relevance and availability, with aorta and heart chosen due to the known link of *Svep1* with cardiovascular disease, kidney selected due to the known role of *Svep1* in blood pressure and to examine the cardio-renal axis, and lung tissue chosen due to the previously reported high expression levels of *Svep1* in the lung. Tissue samples (heart *n* = 15, 9 *Svep1*^*+/−*^ and 6 WT; aorta *n* = 19, 9 *Svep1*^*+/−*^ and 10 WT; lung *n* = 16, 8 *Svep1*^*+/−*^ and 8 WT; kidney n = 16, 8 *Svep1*^*+/−*^ and 8 WT) were removed from storage at −80 °C and kept on dry ice. Samples were washed 3 times in ice cold 50 mM ammonium bicarbonate pH 7.4 + 5 mM EDTA and 1 mL ice cold 1 % ammonium deoxycholate in 50 mM ammonium bicarbonate pH 7.8 + 5 mM EDTA was added to each sample. Samples were homogenized for 3 × 30 s intervals with 5 min resting on ice between each cycle using the BeadBlaster 24 Microtube homogenizer (Benchmark Scientific, New Jersey, USA). Sample homogenates were centrifuged at 14,000*g* at 4 °C for 10 min and the supernatants collected and frozen at −80 °C until required.

### 2.3 In-solution digestion

Aliquots of plasma (*n* = 18, 8 *Svep1*^*+/−*^ and 10 *Svep1*^*+/+*^) and tissue homogenates were defrosted at room temperature. A volume of plasma or tissue homogenate corresponding to 100 μg protein for each sample was determined by bicinchoninic acid assay and aliquoted into a twin.tec 96-well plate (Eppendorf, Hamburg, Germany). Sample positions were randomized across the plate including a pooled quality control sample for each matrix. Samples were made up to 10 μL with 50 mM ammonium bicarbonate pH 7.8. Sample preparation for bottom-up proteomics was performed using the “In-solution Digestion” workflow on the AssayMAP Bravo Platform (Agilent Technologies, Santa Clara, CA, USA). Each step was performed with 15 mix cycles and 3 wash cycles. To the 10 μL starting volume, 20 μL denaturant mixture was added to each sample (2 uL 75 mM dithiothreitol (DTT), 3 μL 1 % rapiGest (Waters, Milford, CT, USA), 15 μL 50 mM ammonium bicarbonate pH 7.8). The plate was sealed and incubated off-deck in a ThermoMixer C (Eppendorf, Hamburg, Germany) at 65 °C for 30 min with agitation at 350 rpm. The plate was returned to the deck and allowed to cool to room temperature, then iodoacetamide (IAA) was added to a final concentration of 10 mM followed by incubation with lid for 30 min. 80 μL ammonium bicarbonate pH 7.8 was added followed by trypsin at a concentration 1:25 protease:protein. The plate was sealed and incubated off-deck at 37 °C for 16 h with agitation at 350 rpm. Samples were acidified with formic acid (FA) 10 % added to a final concentration of 1 % (*v*/v). The plate was centrifuged at 4000 *g* for 45 min using an Eppendorf Centrifuge 5810R (Eppendorf, Hamburg, Germany), and the supernatant collected and transferred into a twin.tec 96-well plate.

### 2.4 Peptide clean-up

Clean-up was performed on the AssayMAP platform following the “Peptide Cleanup” workflow with AssayMAP 5 μL C18 cartridges (Agilent Technologies, Santa Clara, CA, USA) following the default parameters for wash cycles. Cartridges were primed with 100 μL 90 % acetonitrile (MeCN) at a flow rate of 300 μL/min and equilibrated with 50 μL 0.1 % FA at a flow rate of 10 μL/min. 100 μL digests were loaded at a flow rate of 5 μL/min followed by a 25 μL cup wash cycle and 50 μL internal cartridge wash with 0.1 % FA at 10 μL/min. Peptides were eluted with 25 μL 70 % MeCN +0.1 % FA into polypropylene U-bottom 96-well plates (Greiner, Kremsmünster, Austria) at a flow rate of 5 μL/min, dried *in vacuo* and stored at −80 °C until required.

### 2.5 High pH reversed-phase fractionation

Dried plasma samples were resuspended in 100 μL of 25 mM ammonium formate pH 10 and subjected to high pH reversed-phase (RP) fractionation with the AssayMAP Bravo platform using the “Fractionation” workflow with AssayMAP 5 μL RP-S cartridges (Agilent Technologies, Santa Clara, CA, USA). The cartridges were primed with 100 μL 90 % MeCN at a flow rate of 300 μL/min followed by equilibration with 50 μL 25 mM ammonium formate pH 10 at 10 μL/min. Samples were loaded at 5 μL/min followed by a 25 μL cup wash cycle and 50 μL internal cartridge wash with 25 mM ammonium formate pH 10 at 10 μL/min. The peptides were eluted in 7 fractions with 25 mM ammonium formate pH 10, using increasing concentrations of MeCN (5, 10, 15, 20, 30, and 80 %). The fractions were dried *in vacuo* and stored at −80 °C until analysis.

### 2.6 Shotgun liquid chromatography – mass spectrometry

Dried samples were resuspended in 0.1 % FA + 3 % MeCN for LC-MS analysis and peptide concentrations determined by o-phthalaldehyde (OPA) assay to determine peptide concentration such that 500 ng peptide per sample was loaded onto the column for analysis by LC-MS/MS. LC-MS/MS analysis was performed using the Waters nanoAcquity ultra performance LC (UPLC) coupled to the Synapt G2-Si mass spectrometer. Samples were randomized within tissue blocks and baseline instrument performance established using a Pierce commercial HeLa protein digest standard (Thermo Scientific, Loughborough, UK).

The nanoAcquity was equipped with an Acquity UPLC M-Class Symmetry C18 Silica trap column, 100 Å, 5 μm, 20 mm × 180 μm. The analytical column was an Acquity UPLC M-Class HSS T3 column, 100 Å, 1.8 μm, 75 μm × 150 mm. Mobile phase A was H_2_O + 0.1 % and mobile phase B was MeCN +0.1 % FA. The seal wash was H_2_O + 10 % methanol (MeOH), the weak needle wash was H_2_O + 0.1 % FA, and the strong needle wash was MeCN +0.1 % FA. The flow rate was 0.3 μL/min. The autosampler temperature was 8 °C and column temperature was 40 °C. The LC gradient comprised an initial gradient over 65 min from 3 % B to 69 % B and two wash cycles to 90 % B followed by a 7 min re-equilibration to the starting conditions of 3 % B.

The G2-Si was operated in HDMS^E^ data-independent acquisition mode with a Waters Zspray NanoLockSpray in ESI positive mode. The lockspray compound was glu-fibrinogen peptide (GFP) 100 fmol/μL in 50:50 MeCN:H_2_O + 0.1 % FA, measured at an *m/z* of 784.84265. The mass range was set to 50–2000 Da. The ion mobility spectrometry (IMS) wave velocity was 700 m/s and wave height was 15 V. The transfer collision energy was set to 15 V at low energy and at high energy was set as a ramp from 20 to 60 V. The cone voltage was set to 40 V, and the capillary voltage was set to 3.5 kV. Data was acquired using MassLynx V4.2.

### 2.7 Raw data processing

Raw spectral data from the Waters Synapt G2-S were processed label-free using Progenesis QI for Proteomics Software by Nonlinear Dynamics (Waters, Milford, CT, USA) against the reviewed UniProt *mus musculus* database (FASTA format, downloaded June 2023). The digest reagent was set to trypsin with a maximum of 2 missed cleavages. Carbamidomethylation (C) was set as a fixed modification alongside the following variable modifications: oxidation (M), deamidation (N, Q, R), phosphorylation (ST, Y) and acetylation (N-terminus). A false discovery rate (FDR) of 1 % was used with ion matching requirements of 2 fragments per peptide, 5 fragments per protein and 2 peptides per protein. Quantitation was performed using the top N relative quantitation method where *N* = 3. Separate analyses for each fraction was performed for the high pH RP fractionation plasma experiment, which were then recombined to allow for normalisation across fractions. Protein lists with relative abundance data for each identified protein were exported from and Progenesis QI as .csv files.

### 2.8 Statistical analysis and pathway analysis

All statistical analyses were carried out using R version 4.2.0 (The R Foundation for Statistical Computing) on RStudio 2022 (RStudio, Inc., Boston, MA). Each tissue dataset was analysed separately. Data pre-processing was carried out using the R packages NOISeq by Tarazona et al. (2015) [33] and Caret by Kuhn (2008) [34]. First, statistically uninformative features with high numbers of zero values or low variance were filtered from the dataset. The filtered.data function from NOISeq was first used to filter out features which had an average expression per condition of less than or equal to zero. The preProcess function from Caret was used to filter out near zero variance features, removing features in which 90 % of values across samples do not vary. Plotting standard deviation against count data was then used to determine if further filtering was necessary after these steps to remove any additional counts near zero with low variance.

Zero values were then imputed, the data were normalized, and assessed for sources of unwanted technical variation using NOISeq. Run order batch effects were then removed whilst preserving differences between the case/control conditions using the function ARSyNSeq. Normalisation was also carried out using ARSyNSeq, with norm set to “tmm”. This carried out normalisation according to the trimmed mean of M-values method (TMM) [35]. At this stage any zero values are also imputed by replacing each zero with the midpoint between it and the next non-zero value in the matrix.

Differential expression analysis was carried out using the R package Limma by Ritchie et al. (2015) [36]. The linear model was adjusted for the covariates age and sex, and a moderated *t*-test performed incorporating precision weights using voom transformation to stabilize variance across features, Results were filtered for proteins with Benjamini-Hochberg FDR adjusted *p*-values <0.05 and fold change >1.5. The PANTHER (Protein Analysis Through Evolutionary Relationships) v18.0 classification system was used for gene ontology (GO) overrepresentation tests using the GO biological process complete annotation set. [37] STRING (Search Tool for the Retrieval of Interacting Genes/Proteins) v12.0 was used for functional protein association network analysis to predict protein-protein interactions [38]. Clustering of protein interactions was performed using the Markov Cluster (MCL) algorithm.

### 2.9 Stable isotope-labelled internal standard calibration curves

Proteotypic peptides unique to SVEP1 protein within the *mus musculus* proteome were selected using ProteomicsDB proteotypicity rank based on previous measurements by Giansanti et al. in their DDA mass spectrometry-based draft of the mouse proteome [31]: LTCQGNAQWDGPEPR and GAFQQAAQILR. Unlabelled and Stable Isotope-Labelled standards (SILS) were obtained from Peptide Synthetics Peptide Protein Research Ltd. (Hampshire, UK) with a purity of >95 % determined by HPLC. LTC[…] was modified with carbamidomethylation at cysteine. SILS were labelled 13C and 15 N on arginine (C6, N4). Peptides were stored at −20 °C as lyophilized powders until use, whereupon they were solubilized as recommended by the supplier and aliquoted into 100 μL portions for stored at −80 °C until use, whereupon the peptides were diluted to the desired concentration using 0.1 % FA + 3 % MeCN.

Calibration curves were prepared in triplicate using the Andrew+ Pipetting Robot (Waters Andrew Alliance, Milford, CT, USA) and OneLab v1.19.1 software. For each of the SVEP1 peptides (LTC[…] and GAF[…] unlabelled and SILS), fresh stock solutions of 4 pmol/μL were prepared and LTC[…] + GAF[…] combined in a 1:1 ratio to create a separate unlabelled and SILS stock of 4 pmol/μL. The unlabelled peptide mix was 1:2 serially diluted between 1 pmol/μL and 0.5 fmol/μL to give an 11-point calibration curve. A final concentration of 50 fmol/μL SIL added to each calibrator and study samples. A zero calibrator (contains only SILS) and blank (solvent) was also created to assess carryover. Quantitation was performed using the light:heavy peak area ratios based on the sum of the intensities of the y4, y5 and y6 ions. External calibration curves were constructed for the two peptides across five separate analyses in triplicate replicates to validate the accuracy and precision of the assay, with acceptance criteria defined as precision (coefficient of variation, %CV) of 15 % and accuracy (relative error, %RE) within 15 % of the nominal concentration. Positive identification of the analyte in endogenous samples was based on the degree of spectral match between the endogenous peptide and the heavy standard using Skyline's rdotp parameter, where an rdotp of at least 0.9 is preferred, or 0.8 towards the LOD.

### 2.10 Multiple reaction monitoring assay

LC-MS/MS analysis was performed using a Waters Acquity LC coupled to the Xevo TQ-XS mass spectrometer. The LC was equipped with an Acquity Premier Peptide BEH C18 analytical column, 300 Å, 1.7 μm, 2.1 × 50 mm. Mobile phase A was H_2_O + 0.1 % FA. Mobile phase B was acetonitrile (MeCN) + 0.1 % FA. The seal wash was H_2_O + 10 % methanol (MeOH), the weak needle wash was H_2_O + 0.1 % FA, and the strong needle wash was MeCN +0.1 % FA. The flow rate was 0.6 mL/min. The autosampler temperature was 8 °C and column temperature was 40 °C. The Xevo TQ-XS was equipped with a Waters Zspray LockSpray in ESI positive mode. The cone voltage was set to 35 V and the capillary voltage was set to 0.6 kV. The LC gradient comprised a 0.5 min equilibration step at 5 % B, followed by a ramp to 60 % B over 3.2 min, an isocratic hold at 95 % B for 0.9 min, and a re-equilibration to 5 % B for 0.3 mins. Skyline (v 22.2.0.527) [39] with the Prosit deep learning spectral library [40] was used to generate candidate MRM *m/z*. Assay optimization was performed using MassLynx Skyline Interface (MSI) v 1.2.0 for automated selection of optimum transitions, collision energy optimisation, and retention time window scheduling. Precursor and product ion information, optimised collision energies and retention times for each peptide are shown in Table 1. Data was acquired using MassLynx V4.2.

**Table 1:** Sequence and MRM Analysis Details for the two SVEP1 *mus musculus* peptides included in the targeted assay including the precursor *m*/*z*, product ions selected for the analysis, optimised collision energies and retention times.

Peptide Sequence	Precursor charge	Precursor *m/z* (Light/Heavy)	Fragment type	Product ion charge	Product *m/z* (Light/Heavy)	Collision Energy (eV)	Retention Time (mins)
GAFQQAAQILR	2+	601.8/606.8	y7	1+	799.5/809.5	21	1.2
			y6	1+	671.4/681.4	21	
			y3	1+	401.3/411.3	19	
LTCQGNAQWDGPEPR	3+	576.9/580.3	y6	1+	670.3/680.3	15	1.6
			y5	1+	555.3/565.3	21	
			y4	1+	498.3/508.3	17	

## 3 Results

### 3.1 Deep plasma proteome profiling of *Svep1* deficient mice

**Fig. 2:**
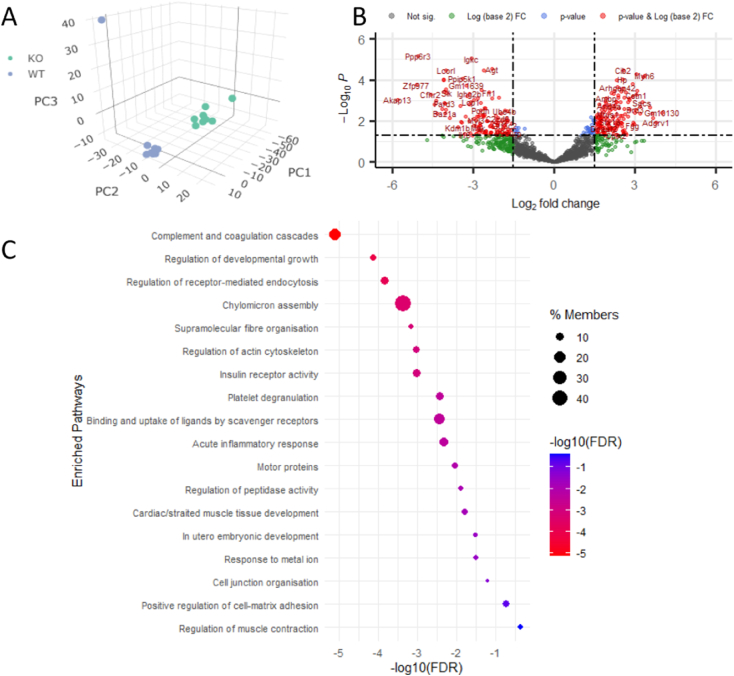
Distinct differences in the plasma proteomes of *Svep1*^*+/−*^ mice (*n* = 8) and WT littermates (*n* = 10). (A) 3D PCA plot demonstrating clear differences in the protein profiles of *Svep1*^*+/−*^ mice (KO, green) compared to wildtype (WT, blue) littermates. Each point represents a single technical (MS) replicate of *n* = 18 KO and WT plasma samples. (B) Volcano plot following differential expression analysis (moderated *t*-test) of *Svep1*^*+/−*^ mice *vs.* WT littermates with vertical cut-offs at FC -1.5 and + 1.5 and horizontal cut-off at BH adj. *p*-value of 0.05. The red features annotated with their accession numbers meet the thresholds: 176 proteins upregulated in *Svep1* deficiency and 136 downregulated. (C) Dot plot showing the enriched biological process pathways for the proteins which are significantly dysregulated in the plasma of *Svep1* deficient mice. For each pathways the adjusted *p*-values (FDR) are shown on the x-axis and colour scale, with the size of the dots representing % Members: the percentage of dysregulated genes in this study that are found in the given ontology term of the pathway. (For interpretation of the references to colour in this figure legend, the reader is referred to the web version of this article.)

We sought to identify differences in the circulating plasma protein signature of *Svep1* deficient mice compared to WT littermates. Owing to the challenges associated with measuring low abundance proteins in undepleted plasma [41], high pH RP fractionation was employed for orthogonal peptide separation prior to RP LC-MS/MS analysis. Each plasma sample (n = 18, 8 *Svep1*^+/−^ and 10 *Svep1*^+/+^) was split into 6 fractions which were analysed separately by LC-MS/MS and recombined and normalized during data processing. 1426 proteins were quantified across the 18 samples. Of note, SVEP1 itself was not identified in this shotgun analysis. Principal component analysis (Fig. 2A) revealed clear discrimination between *Svep1*^+/−^ mice and WT littermates, indicating a downstream difference to the plasma protein profiles of the mice when *Svep1* is knocked out. Differential expression analysis (Fig. 2B) revealed that at Benjamini-Hochberg adjusted *p*-value <0.05 and fold change (FC) of >1.5 or < − 1.5, there were a total of 312 differentially expressed proteins: 176 upregulated in *Svep1* deficiency, and 136 downregulated.

**Fig. 3:**
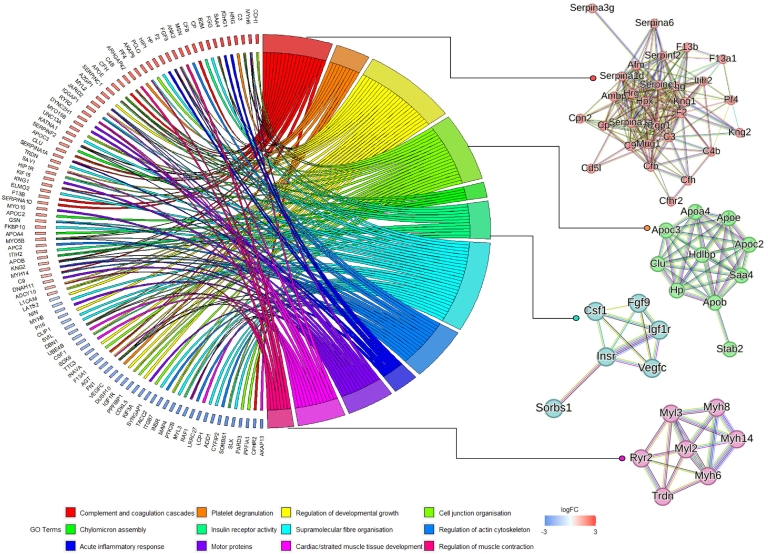
Chord diagram showing the core proteins of 12 significant pathways in the plasma of *Svep1*^*+/−*^ mice (*n* = 8) *vs.* WT littermates (*n* = 10). Significant pathways are shown on the right, connected to their member core proteins on the left with the coloured ribbons. The colour key for each pathways and logFC of the member proteins is shown on the bottom. Fold change of core proteins is shown on the left. On the right, STRING functional protein-protein interaction (PPI) network clusters for a selection of pathways are shown – complement and coagulation cascades (red), chylomicron assembly (green), insulin receptor activity (turquoise), and regulation of muscle development (pink). (For interpretation of the references to colour in this figure legend, the reader is referred to the web version of this article.)

Table 2 shows the 10 most highly up- and downregulated proteins alongside their adjusted *p*-values, log_2_(FC) and functions. All differentially expressed features are shown in Supporting Table S1. For a comprehensive understanding of which pathways were impacted by this change in protein profile, gene ontology functional enrichment analysis was performed using Metascape [42]. The top 20 significantly enriched biological processes at FDR < 0.05 are fully described in Supporting Table S2. The top 5 pathways – complement and coagulation cascades, regulation of developmental growth, receptor-mediated endocytosis, chylomicron assembly and supramolecular fiber organisation – and a selection of others including acute inflammatory response and insulin receptor activity are shown in Fig. 2C. These are also illustrated alongside core proteins from each pathway in Fig. 3.

**Table 2:** Top 10 (based on fold change, FC) significantly upregulated [[1], [2], [3], [4], [5]] and downregulated [[6], [7], [8], [9], [10]] proteins differentially expressed in the plasma of *Svep1*^*+/−*^ mice (*n* = 8) mice and WT littermates (*n* = 10), *i.e.* the proteins top left and top right of the volcano plot shown in Fig. 1B. For each protein, the name, gene symbol, protein accession, adjusted *p*-value (FDR) log2(FC) and a brief synopsis of the protein/gene function is shown.

Protein	GeneID	Accession	FDR	Log2(FC)	Function
Adhesion G-protein coupled receptor V1	ADGRV1	Q8VHN7	0.01	3.8	G-protein coupled receptor with roles in the development of hearing and vision, healthy bone mineral density, and membrane-membrane adhesion.[[13], [14], [15]]
Ring finger protein 213	RNF213	A0A171EBL2	0.007	3.7	Atypical E3 ubiquitin ligase that can catalyse ubiquitination of both proteins and lipids, involved in various processes such as angiogenesis and cell-autonomous immunity, Involved in lipid metabolism by regulating fat storage and lipid droplet formation.[16]
Tubulin tyrosine ligase-like family member 7	TTLL7	A0A0G2LB90	0.004	3.6	Enables tubulin-glutamic acid ligase activity. Involved in protein polyglutamylation.[17]
Cadherin 1	CDH1	A0A0R4IZW5	0.002	3.6	CDH1 plays an essential role in epithelial cell-cell adhesion.[18]
Myosin-6	MYH6	Q02566	0.00006	3.4	Alpha-heavy chain subunit of cardiac myosin involved in cardiac muscle contraction. Acts upstream of or within several processes, including adult heart development; muscle cell development; and regulation of heart contraction.[19]
Translation initiation factor IF-2, mitochondrial	MTIF2	Q91YJ5	0.001058404	−4.3	Predicted to enable GTP binding activity; GTPase activity; and translation initiation factor activity.[20]
Complement factor H-related 2	CFHR2	A0A668KLU9	0.0005	−4.6	Enables complement component C3b binding activity and heparin binding activity. Predicted to be involved in regulation of complement activation. Located in extracellular space.[21]
Protein phosphatase 6 regulatory subunit 3	PPP6R3	G5E8R4	0.000007	−5.1	Regulatory subunit of protein phosphatase 6 (PP6). May function as a scaffolding PP6 subunit. May have an important role in maintaining immune self-tolerance.[22]
Zinc finger protein 977	ZFP977	L7N2E7	0.0002	−5.1	Predicted to enable DNA-binding transcription factor activity.[23]
A-kinase anchor protein 13	AKAP13	E9Q394	0.0009	−5.8	Enables MAP-kinase scaffold activity and Rho GTPase binding.[24,25]

**Fig. 4:**
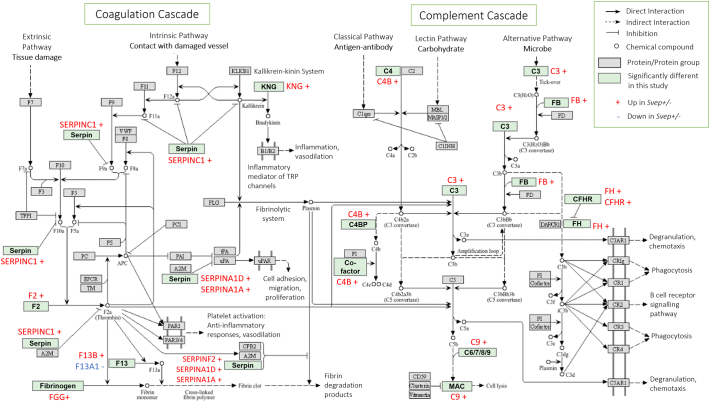
Pathway diagram of complement and coagulation cascades including the Svep1 deficiency-induced differentially expressed proteins. Green boxes show the protein groups for which there are differentially changed components or proteins in this study in the pathway. Proteins in red font are upregulated (+) and in blue font (−) are downregulated. Adapted from KEGG Pathway Maps. (For interpretation of the references to colour in this figure legend, the reader is referred to the web version of this article.)

We sought to further examine the interactome of proteins affected by *Svep1* deficiency by exploring clusters of connected proteins in the STRING functional protein association network [38]. The full connectivity network and is shown in Supporting Fig. S1. There were 62 clusters of interacting proteins identified (33 of which were single proteins or proteins linked to just one other proteins). Within the top 10 clusters with high clustering coefficient were several groups of interacting proteins associated with biological processes also identified as significantly enriched by Metascape (Fig. 3), including: Cluster 1, associated with complement activation; Cluster 2, associated with chylomicron assembly; Cluster 4, associated with heart contraction; Cluster 6, associated with insulin receptor activity. These clusters of interacting proteins have significantly more interactions than would be expected for a random set of proteins of the same size from the genome, indicative that the proteins within each are at least partially biologically connected. The dysregulation of these networks of interacting proteins in *Svep1*^+/−^ mice compared to WT littermates indicates that *Svep1* deficiency has a downstream impact on these connectivity networks and their respective associated pathways in the plasma. The differentially regulated proteins involved in the most enriched pathway, complement and coagulation cascades, are shown on the pathway map for the process in Fig. 4. All proteins involved in this process, with the exception of F13A1, are upregulated indicating upregulation of the complement activation in *Svep1*^*+/−*^ mice compared to controls.

### 3.2 Multi-organ proteomic analysis of *Svep1* deficient mice

We sought to validate and further explore the systemic protein profile changes in the plasma of *Svep1*^*+/−*^ mice in tissue, with a multi-organ proteome analysis comparing the heart, aorta, lung and kidney of *Svep1*^*+/−*^ mice to WT counterparts. Digested tissues (heart *n* = 15, 9 *Svep1*^*+/−*^ and 6 WT; aorta *n* = 19, 9 *Svep1*^*+/−*^ and 10 WT; lung *n* = 16, 8 *Svep1*^*+/−*^ and 8 WT; kidney n = 16, 8 *Svep1*^*+/−*^ and 8 WT) were subjected to LC-MS/MS analysis. 1478 proteins were quantified in aorta, 1681 in heart, 1887 in lung, and 1885 in kidney. SVEP1 was not identified in any of the analyses.

**Fig. 5:**
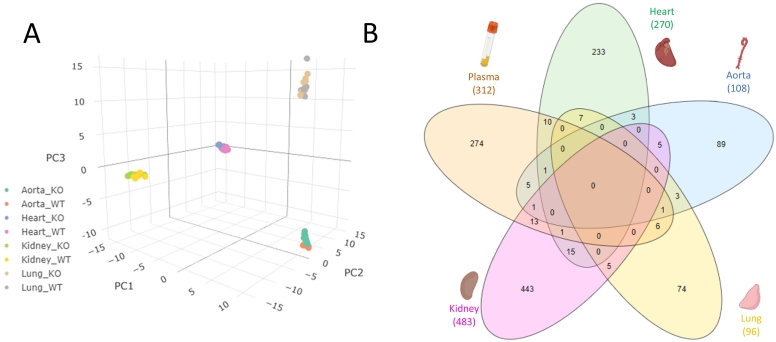
Proteome profiles in the tissue of Svep1+/− mice and WT littermates. Heart *n* = 15, 9 *Svep1*^*+/−*^ and 6 WT; aorta *n* = 19, 9 *Svep1*^*+/−*^ and 10 WT; lung *n* = 16, 8 *Svep1*^*+/−*^ and 8 WT; kidney n = 16, 8 *Svep1*^*+/−*^ and 8 WT. (A) 3D PCA plot demonstrating clear discrimination between each of the mouse tissues (aorta, heart, kidney and lung), and within each tissue different protein profiles between Svep1+/− mice (KO) compared to wildtype (WT) littermates for one technical (MS) replicate of each sample. (B) Venn diagram showing the overlap in dysregulated proteins between the four tissues and plasma. In total there are 1636 differentially expressed proteins in Svep1+/− mice compared to WT littermates. 191 of these proteins are differentially expressed in two or more tissues, 17 in three or more tissues, and 2 proteins in four tissues: Rho GTPase-activating protein 6 (ARHGAP6) and Fibrinogen gamma chain (FGG).

Principal component analysis (Fig. 5A) reveals clear discrimination between each of the tissues, and within each differences in the protein profiles of *Svep1*^*+/−*^ mice and WT littermates. Differential expression analysis revealed that at Benjamini-Hochberg adjusted *p*-value <0.05 and fold change (FC) of >1.5 or < −1.5, there were a total of 270 differentially expressed proteins in heart tissue: 82 upregulated in *Svep1* deficiency and 188 downregulated. In aorta there were 108 differentially expressed proteins: 30 upregulated and 78 downregulated. In lung there were 96 differentially expressed proteins: 16 upregulated and 80 downregulated. In kidney there were 484 differentially expressed proteins: 263 upregulated and 221 downregulated. All differentially expressed features are shown in Supporting Table S3. Volcano plots for each differential expression analysis are shown in Supporting Fig. S2.

**Fig. 6:**
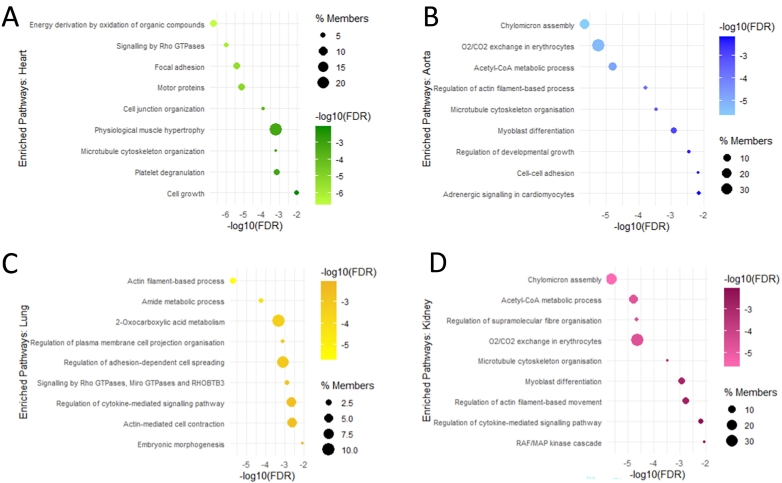
Dot plot showing the enriched biological process pathways for the proteins which are significantly dysregulated in the tissues of Svep1 deficient mice. For each pathways the adjusted *p*-values (FDR) are shown on the x-axis and colour scale, with the size of the dots representing % Members: the percentage of dysregulated genes in this study that are found in the given ontology term of the pathway. Enriched pathways are shown for the (A) Heart (green) (B) Aorta (blue) (C) Lung (yellow) and (D) Kidney (pink). (For interpretation of the references to colour in this figure legend, the reader is referred to the web version of this article.)

**Fig. 7:**
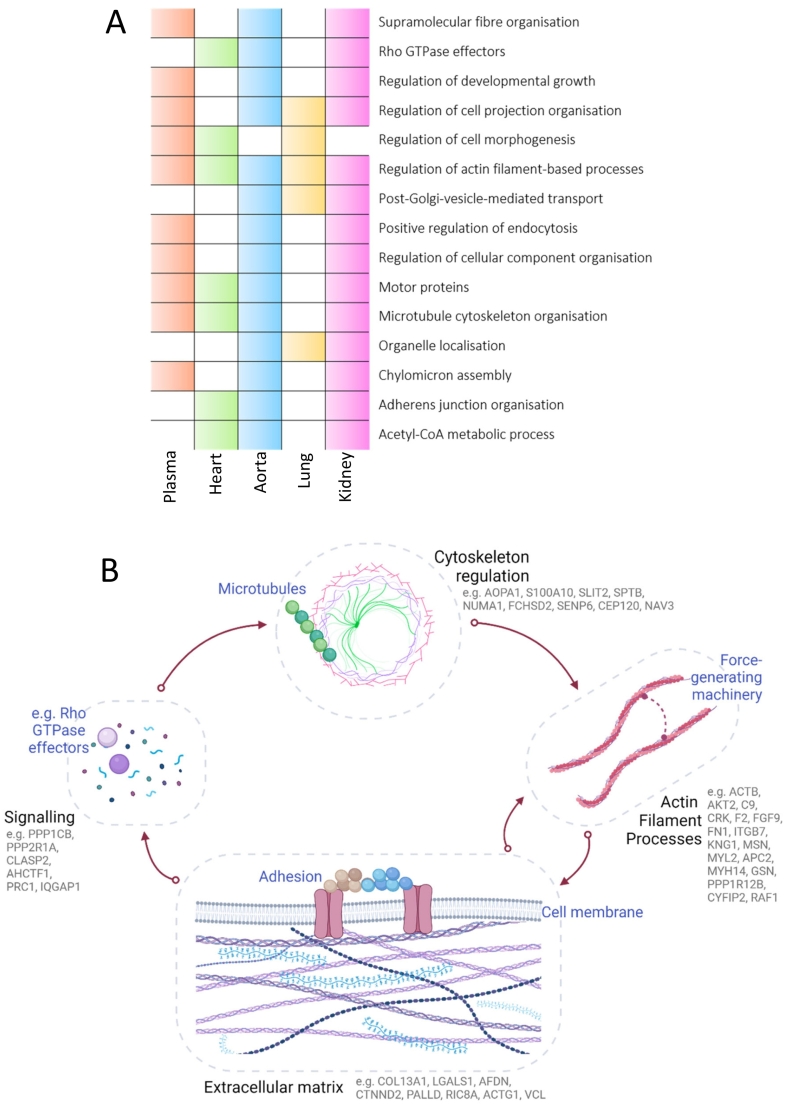
Biological processes which are significantly enriched across tissues and plasma. (A) Overlap in significantly enriched processes across matrices: white-filled space indicates the pathways was not significantly enriched in that particular tissue, and a colour-filled space represents significant enrichment (orange in plasma, green in heart, blue in aorta, yellow in lung, and pink in kidney). (B) Schematic illustrating the interconnected feedback loops between the cytoskeleton, actin machinery, and adhesions at the extracellular matrix. Forces generated by actin polymerisation impact mechanosensitive proteins across multiple functions, including actin-linking, receptor, and co-receptor modules, as well as associated actin-polymerising and signaling modules. This interaction forms a mechanoresponsive network, where the integrated response of the system to matrix interactions and mechanical forces determines the effect on the actin cytoskeleton. Stimulation of the signaling molecules leads to the activation of GTPase-activating proteins, ultimately influencing actin dynamics and the configuration of focal adhesion complexes. Example proteins involved in each process which are differentially expressed in the *Svep1* deficient mice are highlighted. The direction of dysregulation (up/downregulated) for these proteins in each of the tissues can be found in the supporting information. Figure inspired by Geiger et al. [43] and Huber et al. [44]*.* (For interpretation of the references to colour in this figure legend, the reader is referred to the web version of this article.)

Overall, there were 1270 proteins which were dysregulated between the plasma and tissues of *Svep1*^*+/−*^ mice and WT littermates. 76 of these were consistent between two or more tissues, illustrated in Fig. 5B and outlined in Supporting Table S4. 4 proteins were dysregulated in three tissues: carboxylesterase 1C (CES1C), huntingtin-interacting protein 1-related protein (HIP1R), serine protease inhibitor A3G (SERPINA3G), and acetyl-CoA carboxylase 1 (ACACA). For a comprehensive understanding of which pathways were impacted in each tissue and how these compared to the change in plasma, enrichment analysis gene ontology (GO) overrepresentation tests were performed using Metascape [42]. All significantly enriched pathways for each tissue are fully described in Supporting Table S5. The top 5 enriched pathways and up to 4 others at FDR < 0.05 are shown in Fig. 6**.** A number of strong cross-tissue themes were consistently enriched, with 15 pathways which were significantly enriched in three or more tissue types/plasma, shown in Fig. 7. This included regulation of cell projection organisation enriched in each tissue with except heart, and microtubule cytoskeleton organisation and motor proteins, enriched in each except for lung. Actin filament-based processes were enriched in every tissue type and plasma. Other consistent themes emerged around developmental growth and cell morphogenesis, RHO GTPase signaling, chylomicron assembly, and adherens cell junction organisation. Many of these pathways are connected and demonstrate a complex biological interplay; the interaction between actin filament-based processes, cytoskeletal organisation, adhesion and rho GTPases are shown in Fig. 7B.

### 3.3 Targeted LC-MS/MS assay to measure endogenous SVEP1 levels

**Fig. 8:**
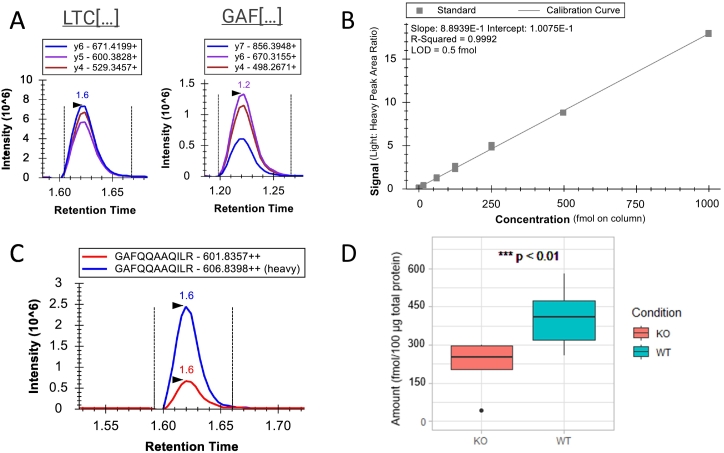
Measurement of SVEP1 using a LC-MS/MS MRM bottom-up proteomics assay. (A) Chromatograms for the unique SVEP1 *mus musculus* peptides LTCQGNAQWDGPEPR (measuring the y6, y4 and y4 fragments) and GAFQQAAQILR (measuring the y7, y6 and y4 fragments), with respective retention times (RT) of 1.6 and 1.2 mins annotated at the peak apex. (B) Ratio-to-heavy calibration curve for determination of the concentration (fmol/μL) of GAF[…]. (C) Chromatogram showing the measurement of endogenous GAF[…] (red, 14.6 fmol) using the ratio to SILS (blue, 50.0 fmol) coeluting at an RT of 1.6 mins (annotated at the peak apex) in a lung homogenate sample. (D) Boxplot showing the levels of SVEP1 GAF[…] measured in the murine lung homogenate (*n* = 16 biological replicates, 8 S*vep1*^*+/−*^ mice *vs.* 8 WT littermates. 3 technical teplicates (MS) per sample). SVEP1 expression is significantly higher in the lungs of WT mice. (For interpretation of the references to colour in this figure legend, the reader is referred to the web version of this article.)

Of note is that in the shotgun LC-MS/MS experiments presented thus far, SVEP1 was not identified in the deep plasma profiling or analysis of tissue homogenates; indeed, the untargeted nature of shotgun LC-MS/MS data acquisition means that the detection of SVEP1 cannot be guaranteed in all experiments. Thus, we aimed to address this by developing a quantitative, targeted multiple reaction monitoring (MRM) LC-MS/MS assay to measure endogenous levels of murine SVEP1 by bottom-up proteomics. A five-minute assay with a limit of detection (LOD) of 0.5 fmol/μL was developed to measure three transitions (precursor-product ion pairs) each for two unique SVEP1 *mus musculus* peptides: LTCQGNAQWDGPEPR and GAFQQAAQILR. Chromatograms for the measurement of two peptide standards at 100 fmol are shown in Fig. 8A. A ratio-to-heavy approach was used for quantitation of the peptides, whereby known quantities of stable isotope-labelled standard (SILS) peptides are spiked into the sample and an external calibration curve constructed to determine the amount of endogenous peptide present by comparing the ratio of peak intensities of the unlabelled peptide and the SILS. External calibration curves were constructed for the two peptides across five separate analyses in triplicate replicates to validate the accuracy and precision of the assay, with acceptance criteria defined in the methods. An exemplar calibration curve for GAF[…] is shown in Fig. 8B, with full accuracy and precision across five separate analyses shown in Supporting Table S6. Based on the better performance of the GAF[…] assay in terms of accuracy and precision, this peptide was selected for quantification of SVEP1, with presence of LTC[…] utilized as a qualifier peptide.

We sought to employ the assay to measure endogenous levels of SVEP1 in plasma, heart, aorta, lung and kidney tissues. SVEP1 was detected in lung, but neither peptide was detected in plasma, heart, aorta or kidney tissues. Chromatograms for the measurement of GAF[…] in an exemplar lung sample are shown in Fig. 8C**,** showing the ratio of the endogenous peptide to the SILS. The assay revealed that the levels of SVEP1 present in lung homogenates of the *Svep1*^*+/−*^ mice was significantly different by Wilcoxon test to that of WT mice, with an average expression level of 7.6 fmol/μL, corresponding to 228 fmol/100 μg total protein, in *Svep*^*+/−*^ and 13.5 fmol/μL, corresponding to 405 fmol/100 μg total protein, in WT mice (Fig. 8D). In ProteomicsDB, median expression of SVEP1 in lung has previously been reported as higher than expression in kidney [31,45] and heart [31,46]. There are no measurements available for SVEP1 protein levels in aorta, however it has previously been found that SVEP1 is more highly expressed in lung than the aorta through quantification of mRNA levels [15]. To our knowledge, no measurements of SVEP1 in the plasma of mice have been performed, with our data suggesting that expression levels in plasma are likely lower than in lung.

## 4 Discussion

Despite strong causal associations with cardiovascular and metabolic disorders including CAD, hypertension, and T2D, as well as a range of other diseases, the exact function of the protein SVEP1 remains largely unknown. In this work, we sought to extensively phenotype the proteome of *Svep1*^+/−^ mice compared to WT littermates with liquid chromatography-tandem mass spectrometry (LC-MS/MS) bottom-up proteomics, in order to infer dysregulated pathways and biological functions associated with *Svep1* deficiency which may be contributing to its role in disease. Across the analysis of plasma and heart, aorta, lung and kidney, *Svep1* deficiency resulted in consistently high numbers of dysregulated proteins, suggesting that deficiency of the *Svep1* gene has extensive downstream impacts on the proteome. The highest numbers of dysregulated proteins were seen in plasma and kidney, and the lowest in lung.

In plasma, some of the most upregulated proteins observed with *Svep1* deficiency shown in Table 2 were ADGRV1, a G protein-coupled receptor with roles in in the development of hearing and vision, healthy bone mineral density, and membrane-membrane adhesion [[47], [48], [49]], CDH1, which plays an essential role in epithelial cell-cell adhesion [50], and MYH6, which is a myofilament protein involved in cardiac muscle contraction [51]. Some of the most downregulated proteins are MTIF2 [52], predicted to enable GTP binding activity and AKAP13, which enables MAP-kinase scaffold activity and Rho GTPase binding [53], Themes around cell adhesion, microtubule cytoskeleton organisation and Rho GTPases were also validated throughout the tissues, as can be seen in Fig. 6. The combination of up- and downregulation within these pathways points to a complex network of dysregulation. These pathways form interconnected feedback loops, shown in Fig. 7B, where stimulation of the signaling molecules leads to the activation of GTPase-activating proteins, ultimately influencing actin dynamics and the configuration of focal adhesion complexes.

The role of SVEP1 in the extracellular matrix and adhesion is well known [14,28,54]. Elenbaas et al. have previously used a proximity-based approach coupled with LC-MS/MS proteomics to elucidate the interactions of SVEP1 in the ECM, finding eight major non-collagen basement membrane components from murine vascular smooth muscle cells (VSMCs) to interact with recombinant SVEP1 [14]. Several of these proteins were found to be downregulated in *Svep1*^*+/−*^ mice in this study, including fibronectin (FN1) in plasma and heart, laminin subunit gamma-1 (LAMC1) in heart and kidney, coatomer subunit alpha (COPA) in lung, and T-complex protein 1 subunit beta (CCT2) in kidney. Ras GTPase-activating-like protein (IQGAP1) was downregulated in both plasma and heart. These findings further support the role of *Svep1* as a cell adhesion protein in the basement membrane linked to the extracellular matrix, with the profile displaying an overall downregulation suggesting a reduction in other basement membrane components in response to *Svep1* deficiency. Additionally, platelet degranulation was enriched in plasma and heart, with a complex network of dysregulation in *Svep1* deficiency including both upregulated (CLU, SERPINF2, PF4, HRG, GFF, KNG), and downregulated (FN1, VEGFC, AHSG, VCL) members. Previous work has found that SVEP1 causally relates to platelet traits [14], and has a role in platelet reactivity [6]. SVEP1 has been found to interact with platelet endothelial aggregation receptor 1 (PEAR1), a receptor tyrosine kinase-like protein which has been found, when phosphorylated, to give rise to extensive platelet degranulation [55]. Thus, SVEP1 deficiency could be giving rise to differential platelet degranulation, perhaps by signaling through PEAR1.

In addition to supporting the known links of *Svep1* to contraction and adhesion, this work has identified potentially novel proteomic signatures associated with *Svep1* deficiency. In plasma, the most significantly enriched pathway was complement and coagulation cascades. SVEP1 is comprised of repeat complement control protein (CCP), or sushi, domains, which control protein interactions including within the complement cascade [56]. As shown on the pathway diagram for complement and coagulation cascades in Fig. 4, upregulation of several key proteins involved throughout the cascade is observed in *Svep1*^+/−^ mice compared to controls including members of the SERPIN family, coagulation factors and fibrinogen. These act in the coagulation cascade leading to cell adhesion, migration and proliferation, platelet activation, vasodilation and anti-inflammatory responses. Several members of the complement cascade are also upregulated including C4B, C3, and C9. This suggests that SVEP1 deficiency leads to an activation of the innate immune response and hemostasis. Indeed, SVEP1 contains a von Willebrand factor type A domain, found in many complement proteins and integrins [57]. Complement protein levels are usually increased during acute or chronic inflammation, thus the overall upregulated profile of complement in response to *Svep1* deficiency, suggests an increased inflammatory response in response to SVEP1 haploinsufficiency. Further validation of this is required, in particular since other studies have found SVEP1 to drive an inflammatory state in atherosclerosis [4].

The insulin receptor signaling activity pathway was enriched in plasma, with key members INSR, VEGFC, IGF1R, CSF1, and SORBS1 (shown in Fig. 3, blue) significantly downregulated in *Svep1*^+/−^ mice compared to WT. Mendelian randomization in human plasma has revealed a causal relationship between SVEP1 and type 2 diabetes [4,5]. Insulin signaling regulates glucose, lipid and energy homeostasis; where signaling pathways are perturbed, this may lead to insulin resistance [58]. Thus, it is plausible that dysregulation of insulin receptor activity could provide a novel mechanism by which *Svep1* mediates T2D, with further validation of this required.

In plasma, aorta and kidney, there was enrichment of pathways associated with chylomicron assembly – triglyceride-rich lipoproteins including fatty acids, and cholesterol. This centered around the dysregulation of an interacting network of apolipoproteins shown in Fig. 3 (green): APOE, APOC2, APOC3, APOC4 and APOB, and other proteins associated with chylomicron clearance SAA4, HDLBP, CLU, HP and STAB2. Given the well-reported connections between SVEP1 and atherosclerosis [4,15], it is plausible that regulation of lipoproteins, complement and coagulation cascades could provide novel mechanisms by which *Svep1* mediates CAD, though further validation of this hypothesis is required. *Svep1* also has positive, causal associations with dementia [13], with lipids playing a complex role in dementia disease pathology, presenting a potential role for the involvement of SVEP1 [59].

An additional hurdle to the study of the contribution of SVEP1 to disease is that reliable measurement of SVEP1 in biological systems can be a difficult task. We aimed to overcome this by developing a high throughput, quantitative targeted multiple reaction monitoring (MRM) LC-MS/MS assay to measure endogenous levels of murine SVEP1. In the shotgun LC-MS/MS experiments presented in this work, SVEP1 was not identified in the deep plasma profiling or analysis of tissue homogenates. A bottom-up proteomics MRM assay with a limit of detection (LOD) of 0.5 fmol was developed and successfully employed to measure SVEP1 in lung homogenate, where SVEP1 expression was significantly lower in *Svep1*^*+/−*^ lung compared to WT. However, the SVEP1 peptides were not detected in plasma, heart, aorta, or kidney. Indeed, it has previously been reported that SVEP1 is more highly expressed in *mus musculus* lung compared to aorta, kidney, and heart [15,31,45] [60], thus it is plausible that SVEP1 was below the limit of detection of the assay in the other tissues. Prior enrichment steps of the SVEP1 peptides with SISCAPA-style anti-peptide antibodies [61] or molecularly imprinted polymers (MIPs) [62] could potentially increase the effective LOD of the assay and allow measurement in these tissues. The transfer of the method to a more sensitive instrument with the use of nanoflow liquid chromatography could also be used to improve LOD of the assay. The mice used in this study were young, healthy mice (8–25 weeks old), and in humans SVEP1 is among the fastest accelerating plasma proteins with age and age-related disease [25], thus expanding the study to include older mice and disease models may also enable measurement of the protein in other tissues and plasma. To our knowledge, the expression levels of SVEP1 protein in mouse plasma have not yet been reported. Conversely, SVEP1 protein expression in human plasma has been measured using various platforms including mass spectrometry and the aptamer-based SomaScan assay platform [3], and is reported to be present at 150 ng/mL according to the Human Plasma PeptideAtlas, well above typical LC-MS/MS limits of detection [63]. If the expression levels are considerably lower in mouse plasma than in that of human plasma, the use of the *Svep1* knockout mouse model may not be representative of the role of circulating SVEP1 in human disease.

Deep phenotyping proteomic assessment has not been extensively conducted in knockout mouse models, and could prove a valuable phenotyping resource to provide a holistic perspective into a variety of knockout models. A small number of previous studies have been performed demonstrating the value of this approach, for example on a hepatic proteomic analysis of selenoprotein T knockout mice [64], suggesting a role in glucose and lipid metabolism. The effects of lipocalin-2 have also been examined in a proteomic study of LNC2 deficient mice [65]. The present study offers a deep phenotyping resource for the study of the function of *Svep1*, providing a broader understanding of the systemic impact of *Svep1* deficiency. LC-MS/MS deep plasma proteome profiling and a multi-organ analysis comprising the heart, aorta, lung and kidney has revealed a complex interplay between *Svep1* and various proteins and pathways, providing insight into potential ways in which the gene influences disease. Further studies should now be performed to validate the relationship of *Svep1* and SVEP1 to potentially novel pathways such as complement and coagulation cascades and chylomicrons. We have also harnessed LC-MS/MS to develop a targeted assay for measurement of murine SVEP1 and provide a novel means to measure the protein in biological systems (specifically lung), though further work should be carried out to improve limits of detection of the assay and examine the levels of SVEP1 in other tissues and especially plasma.

This study was an exploratory study making use of surplus mouse material from other *Svep1* experiments, thus was cost efficient and ethical, however further studies could expand sample size and allow assessment of the impact of age and sex on *Svep1* deficiency. It would be of particular interest to expand the study to older mice and to disease models such as the *Svep1*^*+/−*^*Apoe*^*−/−*^ atheroprone model. Other tissues could also be studied, in particular the brain, liver and eyes. In addition, although the use of a global knockout model to study the effects of *Svep1* deficiency provides valuable insights into the holistic role of SVEP1 across multiple tissues and its interplay with interconnected systemic processes, the use of a global knockout model may introduce confounding factors, such as systemic effects and tissue crosstalk, which could obscure the specific contributions of individual tissues to the observed phenotypes. This underscores the complexity of interpreting global knockout studies more generally. Thus future investigations utilizing tissue-specific knockouts would be useful to pinpoint the contributions of SVEP1 expression in specific tissues to disease phenotypes. Finally, with the use of next generation high resolution LC-MS/MS platforms such as the timsTOF and Orbitrap Astral, future studies could improve proteome depth further to potentially enable measurement of murine SVEP1 in a DIA experiment, and for even more detailed insights into the downstream impact of *Svep1* deficiency on the proteome.

## CRediT authorship contribution statement

**Colleen B. Maxwell:** Writing – review & editing, Writing – original draft, Visualization, Methodology, Investigation, Formal analysis, Data curation, Conceptualization. **Nikita Bhakta:** Software, Methodology, Investigation, Data curation. **Matthew J. Denniff:** Resources, Methodology, Investigation. **Jatinderpal K. Sandhu:** Methodology, Data curation. **Thorsten Kessler:** Resources. **Leong L. Ng:** Writing – review & editing, Supervision. **Donald J.L. Jones:** Writing – review & editing, Supervision. **Tom R. Webb:** Writing – review & editing, Supervision, Resources, Funding acquisition. **Gavin E. Morris:** Writing – review & editing, Supervision, Funding acquisition, Conceptualization.

## Funding sources

This research is funded by the National Institute for Health and Care Research (NIHR) Leicester Biomedical Research Centre (BRC). The views expressed are those of the author(s) and not necessarily those of the NIHR or the Department of Health and Social Care. This work was also supported by the John and Lucille van Geest Foundation.

## Declaration of competing interest

The authors declare they have no competing interests.

## Data Availability

The raw mass spectrometry data generated in this study have been deposited in the MassIVE repository under accession number MSV000095926.

## Appendix A Supplementary data

Supplementary material Image 1
